# Head-to-Head Accuracy Comparison of Three Commercial COVID-19 IgM/IgG Serology Rapid Tests

**DOI:** 10.3390/jcm9082369

**Published:** 2020-07-24

**Authors:** Diego O. Andrey, Patrick Cohen, Benjamin Meyer, Giulia Torriani, Sabine Yerly, Lena Mazza, Adrien Calame, Isabelle Arm-Vernez, Idris Guessous, Silvia Stringhini, Pascale Roux-Lombard, Lionel Fontao, Thomas Agoritsas, Jerôme Stirnemann, Jean-Luc Reny, Claire-Anne Siegrist, Isabella Eckerle, Laurent Kaiser, Nicolas Vuilleumier

**Affiliations:** 1 Division of Laboratory Medicine, Department of Diagnostics, Geneva University Hospitals and Geneva University, 1205 Geneva, Switzerland; patrick.cohen@hcuge.ch (P.C.); sabine.yerly@hcuge.ch (S.Y.); Isabelle.Arm-Vernez@hcuge.ch (I.A.-V.); Pascale.Roux-Lombard@hcuge.ch (P.R.-L.); lionel.fontao@hcuge.ch (L.F.); claire-anne.siegrist@unige.ch (C.-A.S.); laurent.kaiser@hcuge.ch (L.K.); nicolas.vuilleumier@hcuge.ch (N.V.); 2 Division of Infectious Diseases, Department of Medicine, Geneva University Hospitals, 1205 Geneva, Switzerland; lena.mazza@hcuge.ch (L.M.); adrien.calame@hcuge.ch (A.C.); Isabella.Eckerle@hcuge.ch (I.E.); 3 Centre for Vaccinology, Department of Pathology and Immunology, University of Geneva, 1211 Geneva, Switzerland; benjamin.meyer@unige.ch; 4 Department of Microbiology and Molecular Medicine, University of Geneva, 1211 Geneva, Switzerland; giulia.torriani@unige.ch; 5 Division and Department of Primary Care Medicine, Geneva University Hospitals, 1205 Geneva, Switzerland; idriss.guessous@hcuge.ch (I.G.); Silvia.Stringhini@hcuge.ch (S.S.); 6 Unit of Population Epidemiology, Division of Primary Care, Geneva University Hospitals, 1205 Geneva, Switzerland; 7 Division of Immunology and Allergy, Department of Medicine, Geneva University Hospitals, 1205 Geneva, Switzerland; 8 Division of Dermatology, Geneva University Hospitals, 1205 Geneva, Switzerland; 9 Division of General Internal Medicine, Department of Medicine, Geneva University Hospitals, 1205 Geneva, Switzerland; Thomas.Agoritsas@hcuge.ch (T.A.); jerome.stirnemann@hcuge.ch (J.S.); jean-luc.reny@hcuge.ch (J.-L.R.); 10 Department of Childhood and Adolescence, Geneva University Hospitals, 1205 Geneva, Switzerland; 11 Geneva Centre for Emerging Viral Diseases, Geneva University Hospitals and Faculty of Medicine, University of Geneva, 1205 Geneva, Switzerland

**Keywords:** SARS-CoV-2, COVID-19, IgM/IgG serology, rapid test, immunofluorescence, ELISA

## Abstract

Background: Comparative data of SARS-CoV-2 IgM/IgG serology rapid diagnostic tests (RDTs) is scarce. We thus performed a head-to-head comparison of three RDTs. Methods: In this unmatched case-control study, blood samples from 41 RT-PCR-confirmed COVID-19 cases and 50 negative controls were studied. The diagnostic accuracy of three commercially available COVID-19 RDTs: NTBIO (RDT-A), Orient-Gene (RDT-B), and MEDsan (RDT-C), against both a recombinant spike-expressing immunofluorescence assay (rIFA) and Euroimmun IgG ELISA, was assessed. RDT results concordant with the reference methods, and between whole blood and plasma, were established by the Kendall coefficient. Results: COVID-19 cases’ median time from RT-PCR to serology was 22 days (interquartile range (IQR) 13–31 days). Whole-blood IgG detection with RDT-A, -B, and -C showed 0.93, 0.83, and 0.98 concordance with rIFA. Against rIFA, RDT-A sensitivity (SN) was 92% (95% CI: 78–98) and specificity (SP) 100% (95% CI: 91–100), RDT-B showed 87% SN (95% CI: 72–95) and 98% SP (95% CI: 88–100), and RDT-C 100% SN (95% CI: 88–100) and 98% SP (95% CI: 88–100). Against ELISA, SN and SP were above 90% for all three RDTs. Conclusions: RDT-A and RDT-C displayed IgG detection SN and SP above 90% in whole blood. These RDTs could be considered in the absence of routine diagnostic serology facilities.

## 1. Introduction

In the context of COVID-19, numerous CE-marked anti-SARS-CoV-2 serology-based assays have been developed for diagnostics and serological survey purposes, although they are not meant to provide information regarding individual seroprotection statuses [1]. Among them, several rapid diagnostic tests (RDT) have been released for general use, but so far, only a few validation studies assessed their performance against reference methods [2,3,4,5,6,7]. These studies showed varying levels of performances, and cross-comparison is difficult due to: (i) the heterogeneity in the study designs and studied populations (confirmed cases versus seroprevalence studies), (ii) the analytical performance variability, and (iii) only a few head-to-head comparisons between available RDTs

Here, we performed a head-to-head comparison of the performances of three commercially available SARS-CoV-2 IgM/IgG immunochromatographic RDTs, which were provided to the Geneva Reference Centre for Emerging Viral Diseases by the Swiss Red Cross and the Swiss National COVID-19 Science Task Force (https://ncs-tf.ch/en/). These RDTs were from: (i) NTBIO Diagnostics Inc. (Surrey, British Columbia, Canada)—hereafter. RDT-A, (ii) Zhejiang Orient-Gene Biotech Co. Ltd. (Huzhou, China)—hereafter, RDT-B, and (iii) MEDsan GmbH, Biological Health Solutions (Hamburg, Germany)—hereafter, RDT-C.

## 2. Methods

### 2.1. Study Population and Blood Sample Collection

Anonymized leftovers of whole-blood EDTA samples were used for this analysis, in accordance with our institution’s ethical committee and national regulations. We included blood samples from 41 real-time (RT)-PCR confirmed COVID-19 cases hospitalized at the University Hospitals of Geneva, Switzerland and 50 unmatched negative samples from asymptomatic blood donors obtained during the same period (April 2020). Whole-blood EDTA samples were used as a proxy of capillary blood and were centrifuged (3000× *g* for 10 min) in parallel to generate EDTA plasma. All analyses (see below) were performed within 72h of blood sampling without any freeze-thaw cycle. The 41 COVID-19 samples were categorized according to the number of days following real-time (RT)-PCR positivity—days post-diagnosis (DPD).

### 2.2. Study Endpoints

In our primary endpoint, within this cohort of 91 blood samples (45% RT-PCR-confirmed COVID-19), we assessed the accuracy of SARS-CoV-2 IgG detection in whole blood (as a surrogate for capillary blood) by three commercially available IgM/IgG RDTs. This was done by comparing against the recombinant immunofluorescence assay (rIFA) reference method (which identifies IgG targeting the complete SARS-CoV-2 spike protein, i.e., both S1 and S2 subunits).

In our secondary endpoints, we sought to: (i) validate the performances for SARS-CoV-2 IgG detection using the same RDT panel against an ELISA-based IgG serological immunoassay (Euroimmun) as an alternative reference method, (ii) assess the performance of the RDTs against rIFA and IgG ELISA within each of the COVID-19 DPD subgroups (0–14 and >14 days), (iii) assess the result concordances of the three RDTs with both reference methods, (iv) assess the concordance of the rapid IgM/IgG test results in whole blood versus EDTA plasma, and (v) assess IgM detection by each RDT. Due to the absence of a reference comparison method for IgM testing in our institution (not provided by Euroimmun), we did not formally assess RDT IgM detection performances but merely report their positivity ratio within the cohort.

### 2.3. SARS-CoV-2 RT-PCR Analyses

SARS-CoV-2 RT-PCR was performed according to the manufacturer’s instructions on various platforms, including a validated in-house method using eMAG (bioMérieux, Marcy-l’Etoile, France) and the Charité RT-PCR protocol [8] or the BD SARS-CoV-2 reagent kit for the BD Max System (Becton, Dickinson and Co, Franklin Lakes, NJ, USA) or Cobas 6800 SARS-CoV-2 RT-PCR (Roche, Basel, Switzerland).

### 2.4. IgM/IgG Immunochromatographic Rapid Cassette Tests

The three commercially available lateral-flow immunochromatographic SARS-CoV-2 IgM/IgG RDT cassettes can be used with capillary blood, venous whole blood, plasma, or serum. For each tested IgM/IgG RDT, one cassette per sample was used according to the manufacturer’s instructions. For the RDT from NTBIO Diagnostics Inc., Surrey, British Columbia, Canada (hereinafter, RDT-A), 10 µL of whole blood (as a proxy for one capillary blood drop) or 10 µL of plasma were applied in parallel for each sample, and IgG/IgM responses were read after 15 min but no later than 20 min. For the RDT from Zhejiang Orient-Gene Biotech Co. Ltd., Huzhou, China (hereinafter, RDT-B), 10 µL of whole blood or 5 µL of plasma were applied, and results read after 10 min but no later than 15 min. For the RDT from MEDsan GmbH, Biological Health Solutions, Hamburg, Germany (hereinafter, RDT-C), 5 µL of whole blood or 5 µL of plasma were applied, and results read after 10 min but no later than 15 min.

### 2.5. SARS-CoV-2 Spike-Based Recombinant Immunofluorescence Assay

Determination of the IgG antibody response against the complete spike (S) protein (both S1 and S2 domains) of SARS-CoV-2 was performed by recombinant immunofluorescence assay (rIFA), as previously validated for MERS-CoV [9,10] and adapted to SARS-CoV-2 [11]. Briefly, Vero B4 cells, transfected with the SARS-CoV-2 spike protein expression vector pCG1-SCOV2-S (kindly provided by M. Hoffmann and S. Pöhlmann, DPZ, Göttingen, Germany), were seeded on multi-test glass slides, washed, and fixed. After appropriate rehydration and blocking, 30 μL of diluted plasma samples were applied on each spot and incubated for 60 min at 37 °C. Following washing, secondary goat anti-human IgG antibody conjugated with Alexa488 (Jackson ImmunoResearch Europe Ltd., Ely, United Kingdom #109-545-088) was applied to each spot and incubated at 37 °C for 45 min [8]. The rIFA results were assessed by two independent readers with satisfactory interobserver kappa coefficients [11,12,13].

### 2.6. SARS-CoV-2 IgG ELISA

Euroimmun IgG ELISA uses the S1 domain of the spike protein of SARS-CoV-2 as the antigen. EDTA plasma was diluted at 1:101 and assessed with the IgG CE-marked ELISA (Euroimmun AG, Lübeck, Germany # EI 2606-9601 G) according to the manufacturer’s instructions. The ELISAs were run on Dynex Agility (Ruwag, Bettlach, Switzerland) according to the manufacturer’s protocols. After adding the conjugate, samples’ immunoreactivity were measured at an optical density of 450nm (OD450) and then divided by the OD450 of the calibrator provided with each ELISA kit to minimize the inter-assay variation [11,12]. The quantitative results (ratios) obtained were then expressed in arbitrary units and interpreted following the recently published proposed cut-offs derived from our local validation process: OD ratio: < 0.5 = negative, ≥0.5 and <1.5 = indeterminate, and ≥ 1.5 = positive [11,14].

Statistics Vassarstats online tool (www.vassarstats.net) was used to determine the sensitivity (SN), specificity (SP), and positive and negative predictive values (PPV and NPV), as well as the positive and negative likelihood ratios (LR+ and LR−). It was also used to calculate the proportions and 95% confidence intervals. We used PRISM (Graphpad, San Diego, CA, USA) for calculating the medians, interquartile range (IQR), and significance (*p*-values). Significance was calculated using Fisher’s exact test for categorical variables and the Mann-Whitney U-test for continuous variables. Concordance between immunoassays was assessed with Kendall’s coefficient, which was calculated using Statistica (version 13.5.0.17, TIBCO Software Inc., Palo Alto, CA, USA). As previously published, indeterminate rIFA IgG and ELISA IgG results were considered to be negative for the test performances and concordance analyses in order to maximize the specificity [11], as were invalid RDT results. Statistical significance was defined as *p* < 0.05.

## 3. Results

Baseline cohort characteristics. The baseline demographic characteristics of participants were as follows: the 41 RT-PCR-confirmed COVID-19 hospitalized patients were older (median 71 years old, IQR 63–76) compared to the healthy blood donors (median 47 years old, IQR 40–55; *p* = 0.0001). The COVID-19 cohort had a higher proportion of males (*n* = 32, 78.1%) compared to the healthy controls (*n* = 11, 22.0%; *p* = 0.0001). Among the COVID-19 patients, the median delay between a positive SARS-CoV-2 RT-PCR and serology testing was 22 days (IQR 13–31 days). The proportion of patients within each DPD subgroup (delta between the RT-PCR and serological testing) was 34.1% for 0–14 days (*n* = 14) and 65.9% for >14 days (*n* = 27).

rIFA and ELISA IgG seropositivity in RTPCR-confirmed COVID-19 cases and negative controls. Overall, 92.7% (*n* = 38) of RT-PCR confirmed COVID-19 patients had IgG seroconversion detected by rIFA and 90.2% (*n* = 37) by ELISA, as shown in Table 1. In the DPD >14 subgroup, 96.3% (26/27) of COVID-19 patients were seropositive by rIFA. In 49 out of 50 negative controls (98%, 95%CI: 88-100), SARS-CoV-2 IgG could not be detected by rIFA—albeit, one sample was IgG-positive by rIFA and ELISA in both whole blood and plasma (and as well with the three RDTs), suggesting a previously unidentified Covid-19 infection in one of the asymptomatic controls. Detailed results of each test for both COVID-19 and the control cases and samples are available in Supplementary Tables S1–S4.

Performance of the SARS-CoV-2 IgG detection by the RDT panel against rIFA (primary endpoint). Concordance of whole-blood RDT-A, -B, and -C results with rIFA was calculated at 0.93, 0.83, and 0.98, respectively (Kendall correlation coefficient, *p* < 0.0001 for each). Concordance of plasma RDT results with rIFA were slightly higher, as shown in Table 2. The performances of each rapid test for SARS-CoV-2 IgG detection against rIFA are shown in Table 3. For RDT-A in whole blood, we obtained an IgG detection sensitivity (SN) of 92% (95% CI: 78–98) and a specificity (SP) of 100% (95% CI: 91–100%), while the PPV was 100% (95% CI: 88–100) and the NPV was 95% (95% CI: 84–99). RDT-B showed a SN of 87% (95% CI: 72–95) and a SP of 98% (95% CI: 88–100%), while the PPV was 97% (95% CI: 83–100) and the NPV 91% (95% CI: 80–97). Lastly, RDT-C showed a SN of 100% (95% CI: 88–100) and a SP of 98% (95% CI: 88–100%), while the PPV was 98% (95% CI: 85–100) and the NPV was 100% (95% CI: 92–100). Positive likelihood ratios (LR+) were high for all the rapid assays, with an infinite likelihood ratio value for RDT-A, followed by 53 for RDT-C, while LR- were below the nominal value of 0.1 for both. For RDT-B, while the LR+ was 45.33, the LR- was at 0.13. Refer to Table 3 for the rapid serology performances in plasma.

As the secondary endpoint, we analyzed the RDT performances for IgG detection in whole blood across each DPD subgroup and found similar performances in 0-14 DPD and >14 DPD, as shown in Table 3. In DPD 0-14, the SN and SP were above 90% for the three tests. In DPD >14, only RDT-B displayed a SN below 90% at 85% (95% CI: 65–95%).

Performances of SARS-CoV-2 IgG detection by the RDT panel against ELISA. Whole-blood IgG detection result concordances between RDT-A, -B, and -C and Euroimmun ELISA were calculated at 0.91, 0.89, and 0.96, respectively (Kendall orrelation coefficient, *p* < 0.0001 for each) (Table 4). Within this case-control cohort, with a proportion of RT-PCR-confirmed COVID-19 cases of 45.1%, the accuracy of each rapid test against ELISA is shown in Table 4. For RDT-A in whole blood, we obtained an IgG detection SN of 92% (95% CI: 78–98) and a SP of 98% (95% CI: 87–100%), while the PPV was 97% (95% CI: 84–100) and the NPV 95% (95% CI: 84–99). RDT-B showed a SN of 92% (95% CI: 78–98) and a SP of 100% (95% CI: 92–100), while the PPV was 100% (95% CI: 88–100) and the NPV 95% (95% CI: 84–99). Finally, RDT-C showed a SN of 100% (95% CI: 89–100) and a SP of 96% (95% CI: 86–99%), while the PPV was 95% (95% CI: 82–99) and the NPV 100% (95% CI: 91-100). In whole blood, RDT-A showed 48.82 and 0.08 LR+ and LR-, respectively. RDT-B had infinite LR+ and 0.08 LR-. Finally, RDT-C displayed 26.5 and 0.00 LR+ and LR-, respectively. The DPD subgroup analysis showed SN and SP values above 90% and likelihood ratios between infinite LR+ and 0.00 LR-. All values are shown in Table 4, including performances of the RTDs carried out with plasma samples.

SARS-CoV-2 IgG detection by RDTs in whole-blood EDTA versus plasma EDTA. Out of 91 paired samples, result concordances for IgG detection between whole-blood and plasma samples were 0.93, 0.87, and 1.00 for RDT-A, -B, and -C, respectively. The slightly lower concordance observed for RDT-B was influenced by four whole blood–plasma sample pairs, where IgG was detected only in the plasma (Supplementary Table S1). In keeping with these results, RDT-B in plasma showed higher SN (as well as higher NPV and lower negative LR) than in whole blood, as shown in Table 3 and Table 4.

SARS-CoV-2 IgM seropositivity ratios in RT-PCR-confirmed COVID-19 cases and negative controls. IgM detection proportions within these cohorts varied significantly across RDTs, in both whole blood and plasma, ranging from 14.6% positivity within RT-PCR-confirmed COVID-19 samples with RDT-A to 73.2% with RDT-B and 92.7% with RDT-C, as shown in Table 5. In addition, tests performed in plasma rather than whole blood yielded higher positivity for RDT-A and RDT-B, while RDT-C seemed to yield similar results in both sample types. IgM detection result concordances between whole blood and plasma were 0.47, 0.90, and 0.96 for RDT-A, -B, and -C, respectively. Overall, RDT-A showed lower IgM detection capacities and a lower concordance between whole blood and plasma, in favor of the latter. We did not establish here how these differences might affect test performances due to the absence of a reference method for IgM detection (absence of IgM detection by Euroimmun ELISA).

## 4. Discussion

The major finding of the present head-to-head comparison of these SARS-CoV-2 serology RDTs performed on a study sample with a pretest probability of 45.1% is that these RDTs, when carried out in whole blood and compared to rIFA as the primary endpoint, displayed rather acceptable performances overall. Indeed, for the three of them, the SPs were equal or above 98%, and the PPVs ranged between 97% and 100%, keeping in mind that the lowest end of the 95% CI could be as low as 83%. SNs ranged between 87% to 100% and the NPVs between 91% to 100%. When compared to rIFA, RDT-C (MEDsan) appeared to be the most satisfactory of the three RDT tested, with a concordance of 98%, a NPV of 100%, a PPV of 98%, a LR+ of 52, and an optimal LR- of 0.0, although this should be interpreted cautiously due to the rather wide 95% CI. These performances were similar in the two subgroup DPDs below or above 14 days. Regarding the secondary endpoint (association with Euroimmun IgG ELISA) in whole blood, RDT-C (MEDsan) was also found to be close to optimal, with a concordance of 96%, a SN of 100%, a NPV of 100%, a SP of 96%, a PPV of 95%, and an optimal LR- of 0.0, but it also had the lowest LR+ (26.5) among the three RDTs. For this secondary endpoint, when RDTs were used on plasma, RDT-B (Orient-Gene) was found to provide the best concordance with ELISA (98%), the highest LR+ (53), and a close to optimal LR- (0.013). Nevertheless, as RDTs are meant to be used without centrifugation, the implication of this observation is merely technical. Our results contrast with current RDT data available in the literature [2,3,4,5,15,16]. Indeed, Cassaniti and colleagues tested a RDT in the specific setting of acute diagnosis in the emergency department and found very low performance, thus suggesting that RDTs should be avoided in this particular acute clinical setting with potentially very short DPDs. In contrast, a study by Hoffman et al. testing the Zhejiang Orient-Gene RDT revealed a sensitivity of 69% and 93.1% for IgM and IgG, respectively, with very high specificities—although this study compared results against SARS-CoV-2 RT-PCR in the absence of a serology gold standard [2,4]. Thus, data in the literature cannot easily be compared to our results. One major difference between studies is the delay between RT-PCR and serology, which might vary and is generally shorter in other studies. To the best of our knowledge, only this study compared RDT performances against an immunoassay as the reference method.

Several factors can explain the inter-RDT differences. Firstly, it is important to note that the high performances for SARS-CoV-2 IgG detection of the present RDT panel were obtained using a cohort of cases/samples whose median time from RT-PCR to serology testing was approximatively three weeks. This median time was lower in several previous studies. Such a delay allowed to reach high median anti-SARS-CoV2 IgG ratio levels (16.89) measured with Euroimmun ELISA, which, in turn, may explain the good performances reported presently, including in our subgroup analyses in patients belonging to DPD 0-14 versus > 14 DPD, when compared to previous studies performed in acute settings. Secondly, this difference can also be at least partly explained by inter-assay analytical differences, as some assays are focused either against the full spike protein (such as RDT-A), against its subdomains S1 or S2, against the nucleocapsid, or even against a combination thereof (such as RDT-C) [17,18].

The second notable finding of this study lies in the fact that whole-blood EDTA, as a surrogate for capillary blood, is an appropriate medium type for IgG detection with RDT-C (MEDsan) and RDT-A (NTBIO), whereas better performances were obtained with plasma for RDT-B (Orient-Gene). For IgM detection, only RDT-C (MEDsan) showed high positivity rates in whole blood, equal to those in plasma, while plasma seemed to perform better for RDT-A and RDT-B, although further evaluations with an independent reference method is probably needed. Importantly, provided that an adequate RDT is used, these results suggest that whole blood can be an adequate medium for SARS-CoV-2 IgG serology testing. Of note, despite the large variability observed across RDTs for IgM detection, RDT-C (MEDsan) seemed to display the highest detection rate.

A limitation to this study is, firstly, that we used whole blood as a proxy for capillary blood, and the test was performed in a laboratory environment; although whole blood EDTA is an established surrogate of capillary blood [19], we would possibly expect different results in real life with a non-laboratory-trained individual using capillary blood. Secondly, this is a method validation study and not a seroprevalence study. The case-control design, with a final proportion of RT-PCR-confirmed COVID-19 cases of 45.1%, warrants a cautious interpretation of the PPV and NPV. Nevertheless, given the very high and low LR+ and LR-, respectively, our results indicate that that these RDT may prove to be useful even in lower pretest probability settings, where the current findings may provide guidance on the choice of the RDT to be considered. Third, within the asymptomatic control group, in one sample, all tested immunoassays (the three RDTs and both reference methods) yielded positive results. Although it did not influence the test performance analysis, it suggests a possible SARS-CoV-2 serological scar within the control population of the blood donors. When selecting healthy controls, we had no way to avoid the inclusion of a subject with a previous, possibly asymptomatic, infection during the epidemic period. Fourthly, the limited sample size of this validation study leads to broad 95% confidence intervals, precluding a robust ranking of the three RDTs under study. Finally, among all the RDTs available on the market, we only tested three of them based upon the prior selection made by the Swiss Red Cross and the Swiss COVID-19 Task Force. Therefore, our present conclusions only apply to these RDTs and must not be applied to any other RDTs. Taken together, these data indicate that RDTs should probably not be used if access to centralized tests is available [20].

In conclusion, among the three RDTs tested, the one from MEDsan (RDT-C) in whole blood displayed the highest concordance with our reference method (rIFA) and with automated ELISA, with close to optimal positive and negative predictive values in this high pretest probability cohort. Even if these RDT are currently not meant to replace centralized tests and should not be used whenever routine laboratory-based SARS-CoV-2 IgG serology is available, the apparently adequate performance of this RDT merits further testing in lower prevalence settings.

## Figures and Tables

**Table 1:** SARS-CoV-2 IgG detection characteristics by various immunoassays.

Samples	No (%)	rIFA IgG Positive No (%)	ELISA IgG		RDT-A IgG Positive No (%)		RDT-B IgG Positive No (%)		RDT-C IgG Positive No (%)	
			Positive No (%)	Ratio Median (IQR) ^a^	WB-EDTA	plasma-EDTA	WB-EDTA	plasma-EDTA	WB-EDTA	plasma-EDTA
Negative controls	50 (100)	1 (2)	1 (2)	0.30 (0.27–0.42)	1 (2)	1 (2)	1 (2)	1 (2)	1 (2)	1 (2)
COVID-19										
All	41 (100)	38 (92.7)	37 (90.2)	16.89 (11.56–18.15)	36 (87.8)	36 (87.8)	34 (85.0) ^b^	38 (92.7)	39 (95.1)	39 (95.1)
DPD 0–14	14 (34.1)	12 (85.7)	13 (92.9)	16.04 (8.17–18.02)	11 (78.6)	11 (78.6)	12 (85.7)	13 (92.9)	13 (92.9)	13 (92.9)
DPD > 14	27 (65.9)	26 (96.3)	24 (88.9)	17.06 (14.53–18.65)	25 (92.6)	25 (92.6)	22 (84.6) ^b^	25 (92.6)	26 (96.3)	26 (96.3)

^a^ Cut-offs < 0.5 = negative, ≥ 0.5 and < 1.5 = indeterminate, and ≥ 1.5 = positive. ^b^ Due to one invalid test, the denominator is 40 positive samples DPD (days post-diagnosis). rapid diagnostic test (RDT)-A, NTBIO; RDT-B, Orient-Gene; and RDT-C, MEDsan Rapid Diagnostic Test. WB, whole blood and rIFA, recombinant immunofluorescence assays. IQR, interquartile range.

**Table 2:** Correlation between RDT versus rIFA and ELISA results.

Kendall τ Coefficient ^a^ *N* = 91 Pairs		rIFA IgG	ELISA IgG
RDT-A IgG	WB	0.93	0.91
	Plasma	0.96	0.89
RDT-B IgG	WB	0.83	0.89
	Plasma	0.96	0.98
RDT-C IgG	WB	0.98	0.96
	Plasma	0.98	0.96

^a^ All *p*-values < 0.0001. RDT-A, NTBIO; RDT-B, Orient-Gene; RDT-C, MEDsan Rapid Diagnostic Test; WB, whole blood; and rIFA, recombinant immunofluorescence assays.

**Table 3:** RDT performances against rIFA.

		SN (95CI) %	SP (95CI) %	PPV (95CI) %	NPV (95CI) %	LR+ (95CI) %	LR- (95CI) %
All cases (*n* = 91) versus rIFA	RDT-A WB	92 (78–98)	100 (91–100)	100 (88–100)	95 (84–99)	∞	0.08 (0.03–0.23)
	RDT-A plasma	95 (81–99)	100 (91–100)	100 (88–100)	96 (86–99)	∞	0.05 (0.01–0.20)
	RDT-B WB	87 (72–95)	98 (88–100)	97 (83–100)	91 (80–97)	45.33 (6.48–316.97)	0.13 (0.06–0.30)
	RDT-B plasma	97 (85–100)	98 (88–100)	97 (85–100)	98 (88–100)	50.67 (7.27–353.17)	0.03 (0.00–0.18)
	RDT-C WB	100 (88–100)	98 (88–100)	98 (85–100)	100 (92–100)	52.00 (7.46–362.23)	0.00
	RDT-C plasma	100 (88–100)	98 (88–100)	98 (85–100)	100 (92–100)	52.00 (7.46–362.23)	0.00
DPD 0-14 and controls (*n* = 64) versus rIFA ^a^	RDT-A	92 (62–100)	100 (91–100)	100 (70–100)	98 (88–100)	∞	0.08 (0.01–0.51)
	RDT-B	92 (62–100)	98 (88–99)	92 (62–100)	98 (88–100)	47.08 (6.72–329.89)	0.08 (0.01–0.52)
	RDT-C	100 (72–100)	98 (88–100)	93 (64–100)	100 (91–100)	51.00 (7.32–355.13)	0.00
DPD > 14 and controls (*n* = 77) versus rIFA ^a^	RDT-A	93 (74–99)	100 (91–100)	100 (83–100)	96 (86-99)	∞	0.07 (0.02–0.28)
	RDT-B	85 (65–95)	100 (91–100)	100 (82–100)	93 (81-98)	∞	0.15 (0.06–0.37)
	RDT-C	100 (85–100)	100 (91–100)	100 (85–100)	100 (91-100)	∞	0.00

^a^ Only whole-blood (WB) samples were analyzed in the DPD sub-analysis. DPD, days post-diagnosis; Sn, sentivity; Sp, specificity; PPV, positive predictive value; NPV, negative predicitve value; LR+, positive likelihood ratio; LR-, negative likelihood ratio; RDT-A, NTBIO; RDT-B, Orient-Gene, RDT-C, MEDsan Rapid Diagnostic Test; rIFA, recombinant immunofluorescence assay; and ∞, infinite.

**Table 4:** RDT performances against ELISA.

		SN (95CI) %	SP (95CI) %	PPV (95CI) %	NPV (95CI) %	LR+ (95CI) %	LR-(95CI) %
All cases (n = 91) versus ELISA	RDT-A WB	92 (78–98)	98 (89–100)	97 (84–100)	95 (84–99)	48.82 (6.99-340-93)	0.08 (0.03–0.24)
	RDT-A plasma	92 (78–98)	96 (86–99)	95 (81–99)	94 (84–99)	24.41 (6.25-95.35)	0.08 (0.03–0.24)
	RDT-B WB	92 (78–98)	100 (92–100)	100 (88–100)	95 (84–99)	∞	0.08 (0.03–0.23)
	RDT-B plasma	100 (89–100)	98 (89–100)	97 (85–100)	100 (91–100)	53.00 (7.61-369.32)	0.00
	RDT-C WB	100 (89–100)	96 (86–99)	95 (82–99)	100 (91–100)	26.50 (6.81-103.20)	0.00
	RDT-C plasma	100 (89–100)	96 (86–99)	95 (82–99)	100 (91–100)	26.50 (6.81–103.20)	0.00
DPD 0-14 and controls (n = 64) versus ELISA ^a^	RDT-A	86 (56–98)	100 (91–100)	100 (70–100)	96 (86–99)	∞	0.14 (0.04–0.52)
	RDT-B	93 (64–100)	100 (91–100)	100 (72–100)	98 (88–100)	∞	0.07 (0.01–0.47)
	RDT-C	100 (73–100)	100 (91–100)	100 (73–100)	100 (91–100)	∞	0.00
DPD >14 and controls (n = 77) versus ELISA ^a^	RDT-A	96 (78–100)	98 (88–100)	96 (78–100)	98 (88–100)	49.92 (7.16–348.31)	0.04 (0.01–0.28)
	RDT-B	92 (73–99)	100 (91–100)	100 (82–100)	96 (86–99)	∞	0.08 (0.02–0.30)
	RDT-C	100 (83–100)	96 (86–99)	93 (74–99)	100 (91–100)	26 (6.68–101.20)	0.00

^a^ Only whole-blood (WB) samples were analyzed in the DPD sub-analysis. DPD, days post-diagnosis; Sn, sentivity; Sp, specificity; PPV, positive predictive value; NPV, negative predicitve value; LR+, positive likelihood ratio; LR-, negative likelihood ratio; RDT-A, NTBIO; RDT-B, Orient-Gene, RDT-C, MEDsan Rapid Diagnostic Test; rIFA, recombinant immunofluorescence assay; and ∞, infinite LR+.

**Table 5:** SARS-CoV-2 IgM detection characteristics by various immunoassays.

Samples	No (%)	RDT-A IgM Positive No (%)		RDT-B IgM Positive No (%)		RDT-C IgM Positive No (%)	
		WB-EDTA	plasma-EDTA	WB-EDTA	plasma-EDTA	WB-EDTA	plasma-EDTA
Negative controls	50 (100)	0	0	1 (2)	1 (2)	1 (2)	1 (2)
COVID-19							
All	41 (100)	6 (14.6)	10 (24.4)	30 (73.2)	34 (82.9)	38 (92.7)	38 (92.7)
DPD 0–14	14 (34.1)	3 (21.4)	4 (28.6)	10 (71.4)	12 (85.7)	13 (92.9)	12 (85.7)
DPD > 14	27 (65.9)	3 (11.1)	6 (22.2)	20 (74.1)	22 (81.5)	25 (92.6)	26 (96.3)

DPD, days post-diagnosis; RDT-A, NTBIO; RDT-B, Orient-Gene, RDT-C, MEDsan Rapid Diagnostic Test; and WB, whole blood.

## Supplementary Materials

The following are available online at https://www.mdpi.com/2077-0383/9/8/2369/s1, Table S1. Characteristics of SARS-CoV-2 IgG detection by three COVID-19 IgM/IgG Rapid Test, recombinant IFA and Euroimmun IgG ELISA immunoassays in 41 hospitalized qRT-PCR confirmed COVID-19 patients; Table S2. Characteristics of SARS-CoV-2 IgM detection by three COVID-19 IgM/IgG Rapid Test in 41 hospitalized qRT-PCR confirmed COVID-19 patients; Table S3. Characteristics of SARS-CoV-2 IgG detection by three COVID-19 IgM/IgG Rapid Test, recombinant IFA and Euroimmun IgG ELISA immunoassays in 50 asymptomatic controls; Table S4. Characteristics of SARS-CoV-2 IgM detection by three COVID-19 IgM/IgG RDT in 50 asymptomatic controls.
