# Supplementary material for: Head-to-Head Accuracy Comparison of Three Commercial COVID-19 IgM/IgG Serology Rapid Tests
Source: J Clin Med. 2020 Jul 24;9(8):2369. doi: 10.3390/jcm9082369 (PMC7463984; doi:10.3390/jcm9082369)
Supplement: Supplementary file 1 [file jcm-09-02369-s001.pdf]

SUPPLEMENTARY MATERIALS

Table S1. Characteristics of SARS-CoV-2 IgG detection by three COVID-19 IgM/IgG Rapid Test, recombinant IFA and Euroimmun IgG ELISA immunoassays in 41 hospitalized qRT-PCR confirmed COVID-19 patients.

Case	Sex	Age	DPD (Days)	IgG RDT-A		IgG RDT-B		IgG RDT-C		rIFA IgG	ELISA IgG	
				Whole blood EDTA	Plasma EDTA	Whole blood EDTA	Plasma EDTA	Whole blood EDTA	Plasma EDTA		Ratio	Result
1	f	74	29	POS	POS	POS	POS	POS	POS	POS	15.39	POS
2	m	57	9	POS	POS	POS	POS	POS	POS	POS	14.59	POS
3	f	83	19	NEG	NEG	NEG	NEG	NEG	NEG	NEG	0.21	NEG
4	m	56	23	POS	POS	POS	POS	POS	POS	POS	16.62	POS
5	m	53	10	NEG	NEG	POS	POS	POS	POS	NEG	2.98	POS
6	f	63	24	POS	POS	POS	POS	POS	POS	POS	18.21	POS
7	m	71	31	POS	POS	POS	POS	POS	POS	POS	16.32	POS
8	m	76	22	NEG	POS	NEG	POS	POS	POS	POS	0.53	INDET.
9	m	80	24	POS	POS	POS	POS	POS	POS	POS	15.77	POS
10	f	82	9	NEG	NEG	NEG	POS	POS	POS	POS	8.58	POS
11	m	50	0	POS	POS	POS	POS	POS	POS	POS	18.41	POS
12	m	76	13	POS	POS	POS	POS	POS	POS	POS	6.95	POS
13	f	74	9	NEG	NEG	NEG	NEG	NEG	NEG	NEG	0.47	NEG
14	f	60	26	POS	POS	POS	POS	POS	POS	POS	17.75	POS
15	m	75	34	POS	POS	POS	POS	POS	POS	POS	18	POS
16	m	70	9	POS	POS	POS	POS	POS	POS	POS	15.8	POS
17	m	66	34	POS	POS	POS	POS	POS	POS	POS	17.91	POS
18	m	78	30	POS	POS	POS	POS	POS	POS	POS	14.53	POS
19	f	76	31	POS	POS	INVALID	POS	POS	POS	POS	2.04	POS
20	m	66	36	POS	POS	POS	POS	POS	POS	POS	17.84	POS
21	m	74	13	POS	POS	POS	POS	POS	POS	POS	16.89	POS
22	f	60	14	POS	POS	POS	POS	POS	POS	POS	14.83	POS
23	m	79	9	POS	POS	POS	POS	POS	POS	POS	18	POS
24	m	68	30	POS	POS	POS	POS	POS	POS	POS	18.92	POS
25	m	51	13	POS	POS	POS	POS	POS	POS	POS	18.08	POS
26	m	66	25	POS	POS	POS	POS	POS	POS	POS	18.96	POS
27	m	67	31	POS	NEG	POS	POS	POS	POS	POS	18.89	POS
28	m	78	35	POS	POS	NEG	POS	POS	POS	POS	18.65	POS
29	f	84	19	POS	POS	POS	POS	POS	POS	POS	2.16	POS
30	m	64	28	POS	POS	POS	POS	POS	POS	POS	17.94	POS
31	m	76	22	POS	POS	POS	POS	POS	POS	POS	16.91	POS
32	m	51	28	POS	POS	POS	POS	POS	POS	POS	20.64	POS
33	m	83	14	POS	POS	POS	POS	POS	POS	POS	17.03	POS
34	m	62	15	POS	POS	NEG	NEG	POS	POS	POS	0.59	INDET .
35	m	65	41	POS	POS	POS	POS	POS	POS	POS	17.06	POS

36	m	81	11	POS	POS	POS	POS	POS	POS	POS	19.86	POS
37	m	70	38	POS	POS	POS	POS	POS	POS	POS	15.61	POS
38	m	71	16	POS	POS	POS	POS	POS	POS	POS	19.82	POS
39	m	72	14	POS	POS	POS	POS	POS	POS	POS	16.27	POS
40	m	71	16	NEG	POS	POS	POS	POS	POS	POS	6.16	POS
41	m	56	35	POS	POS	POS	POS	POS	POS	POS	18.66	POS

Table S2. Characteristics of SARS-CoV-2 IgM detection by three COVID-19 IgM/IgG Rapid Test in 41 hospitalized qRT-PCR confirmed COVID-19 patients.

Case	Sex	Age	DPD (Days)	IgM RDT-A		IgM RDT-B		IgM RDT-C	
				Whole blood	Plasma EDTA	Whole blood	Plasma EDTA	Whole blood	Plasma EDTA
1	f	74	29	NEG	NEG	POS	POS	POS	POS
2	m	57	9	NEG	NEG	POS	POS	POS	POS
3	f	83	19	NEG	NEG	NEG	NEG	NEG	NEG
4	m	56	23	NEG	NEG	POS	POS	POS	POS
5	m	53	10	NEG	NEG	POS	POS	POS	POS
6	f	63	24	NEG	NEG	POS	POS	POS	POS
7	m	71	31	NEG	NEG	NEG	NEG	POS	POS
8	m	76	22	NEG	NEG	NEG	NEG	POS	POS
9	m	80	24	POS	NEG	POS	POS	POS	POS
10	f	82	9	NEG	NEG	NEG	NEG	POS	NEG
11	m	50	0	NEG	NEG	POS	POS	POS	POS
12	m	76	13	NEG	NEG	NEG	POS	POS	POS
13	f	74	9	NEG	NEG	NEG	NEG	NEG	NEG
14	f	60	26	NEG	NEG	POS	POS	POS	POS
15	m	75	34	NEG	NEG	NEG	POS	POS	POS
16	m	70	9	NEG	POS	POS	POS	POS	POS
17	m	66	34	NEG	POS	POS	POS	POS	POS
18	m	78	30	NEG	NEG	POS	POS	POS	POS
19	f	76	31	NEG	NEG	INVALID	NEG	POS	POS
20	m	66	36	NEG	NEG	POS	POS	POS	POS
21	m	74	13	NEG	NEG	NEG	POS	POS	POS
22	f	60	14	POS	POS	POS	POS	POS	POS
23	m	79	9	NEG	NEG	POS	POS	POS	POS
24	m	68	30	NEG	NEG	POS	POS	POS	POS
25	m	51	13	POS	POS	POS	POS	POS	POS
26	m	66	25	NEG	NEG	NEG	POS	POS	POS
27	m	67	31	POS	POS	POS	POS	POS	POS
28	m	78	35	NEG	NEG	POS	POS	POS	POS
29	f	84	19	NEG	NEG	POS	POS	POS	POS
30	m	64	28	NEG	POS	POS	POS	POS	POS
31	m	76	22	NEG	NEG	POS	POS	POS	POS
32	m	51	28	NEG	POS	POS	POS	POS	POS
33	m	83	14	NEG	NEG	POS	POS	POS	POS
34	m	62	15	NEG	POS	NEG	NEG	POS	POS
35	m	65	41	POS	POS	POS	POS	POS	POS

36	m	81	11	POS	NEG	POS	POS	POS	POS
37	m	70	38	NEG	NEG	POS	POS	POS	POS
38	m	71	16	NEG	NEG	POS	POS	POS	POS
39	m	72	14	NEG	POS	POS	POS	POS	POS
40	m	71	16	NEG	NEG	POS	POS	NEG	POS
41	m	56	35	NEG	NEG	POS	POS	POS	POS

Table S3. Characteristics of SARS-CoV-2 IgG detection by three COVID-19 IgM/IgG Rapid Test, recombinant IFA and Euroimmun IgG ELISA immunoassays in 50 asymptomatic controls

Case	Sex	Age	IgG RDT-A		IgG RDT-B		IgG RDT-C		rIFA IgG	ELISA IgG	
			WB	Plasma	WB	Plasma	WB	Plasma		Ratio	Result
1	f	55	NEG	NEG	NEG	NEG	NEG	NEG	NEG	0.67	INDET.
2	m	24	NEG	NEG	NEG	NEG	NEG	NEG	NEG	0.52	INDET.
3	f	48	NEG	NEG	NEG	NEG	NEG	NEG	NEG	0.34	NEG
4	f	52	NEG	NEG	NEG	NEG	NEG	NEG	NEG	0.43	NEG
5	m	47	NEG	NEG	NEG	NEG	NEG	NEG	NEG	0.42	NEG
6	f	30	NEG	NEG	NEG	NEG	NEG	NEG	NEG	0.31	NEG
7	m	36	NEG	NEG	NEG	NEG	NEG	NEG	NEG	0.63	INDET.
8	f	47	NEG	NEG	NEG	NEG	NEG	NEG	NEG	0.85	INDET.
9	m	42	NEG	NEG	NEG	NEG	NEG	NEG	NEG	0.35	NEG
10	f	59	NEG	NEG	NEG	NEG	NEG	NEG	NEG	0.34	NEG
11	m	29	NEG	NEG	NEG	NEG	NEG	NEG	NEG	0.36	NEG
12	f	42	NEG	NEG	NEG	NEG	NEG	NEG	NEG	0.29	NEG
13	f	47	NEG	NEG	NEG	NEG	NEG	NEG	NEG	0.34	NEG
14	f	53	NEG	NEG	NEG	NEG	NEG	NEG	NEG	0.29	NEG
15	f	47	NEG	NEG	NEG	NEG	NEG	NEG	NEG	0.22	NEG
16	m	48	NEG	NEG	NEG	NEG	NEG	NEG	NEG	0.41	NEG
17	f	61	POS	POS	POS	POS	POS	POS	POS	2.81	POS
18	m	49	NEG	NEG	NEG	NEG	NEG	NEG	NEG	0.26	NEG
19	f	30	NEG	NEG	NEG	NEG	NEG	NEG	NEG	0.26	NEG
20	f	59	NEG	NEG	NEG	NEG	NEG	NEG	NEG	0.29	NEG
21	f	35	NEG	NEG	NEG	NEG	NEG	NEG	NEG	0.25	NEG
22	f	43	NEG	NEG	NEG	NEG	NEG	NEG	NEG	0.3	NEG
23	f	61	NEG	NEG	NEG	NEG	NEG	NEG	NEG	0.33	NEG
24	f	55	NEG	NEG	NEG	NEG	NEG	NEG	NEG	0.2	NEG
25	m	38	NEG	NEG	NEG	NEG	NEG	NEG	NEG	0.61	INDET.
26	f	46	NEG	NEG	NEG	NEG	NEG	NEG	NEG	0.27	NEG
27	f	48	NEG	NEG	NEG	NEG	NEG	NEG	NEG	0.24	NEG
28	f	22	NEG	NEG	NEG	NEG	NEG	NEG	NEG	0.28	NEG
29	f	61	NEG	NEG	NEG	NEG	NEG	NEG	NEG	0.25	NEG
30	f	22	NEG	NEG	NEG	NEG	NEG	NEG	NEG	0.26	NEG
31	f	44	NEG	NEG	NEG	NEG	NEG	NEG	NEG	0.47	NEG
32	f	58	NEG	NEG	NEG	NEG	NEG	NEG	NEG	0.3	NEG
33	m	46	NEG	NEG	NEG	NEG	NEG	NEG	NEG	0.25	NEG
34	f	40	NEG	NEG	NEG	NEG	NEG	NEG	NEG	0.26	NEG
35	f	61	NEG	NEG	NEG	NEG	NEG	NEG	NEG	0.29	NEG
36	f	37	NEG	NEG	NEG	NEG	NEG	NEG	NEG	0.27	NEG
37	f	44	NEG	NEG	NEG	NEG	NEG	NEG	NEG	0.29	NEG
38	f	55	NEG	NEG	NEG	NEG	NEG	NEG	NEG	0.31	NEG

39	f	50	NEG	NEG	NEG	NEG	NEG	NEG	NEG	0.46	NEG
40	f	39	NEG	NEG	NEG	NEG	NEG	NEG	NEG	0.28	NEG
41	f	49	NEG	NEG	NEG	NEG	NEG	NEG	NEG	0.31	NEG
42	f	46	NEG	NEG	NEG	NEG	NEG	NEG	NEG	0.28	NEG
43	f	62	NEG	NEG	NEG	NEG	NEG	NEG	NEG	0.27	NEG
44	f	46	NEG	NEG	NEG	NEG	NEG	NEG	NEG	0.78	INDET.
45	f	62	NEG	NEG	NEG	NEG	NEG	NEG	NEG	0.43	NEG
46	f	60	NEG	NEG	NEG	NEG	NEG	NEG	NEG	0.27	NEG
47	f	46	NEG	NEG	NEG	NEG	NEG	NEG	NEG	0.25	NEG
48	m	55	NEG	NEG	NEG	NEG	NEG	NEG	NEG	0.35	NEG
49	m	57	NEG	NEG	NEG	NEG	NEG	NEG	NEG	0.3	NEG
50	f	35	NEG	NEG	NEG	NEG	NEG	NEG	NEG	0.81	INDET.

Table S4. Characteristics of SARS-CoV-2 IgM detection by three COVID-19 IgM/IgG RDT in 50 asymptomatic controls

Case	Sex	Age	IgG RDT-A		IgG RDT-B		IgG RDT-C	
			WB	Plasma	WB	Plasma	WB	Plasma
1	f	55	NEG	NEG	NEG	NEG	NEG	NEG
2	m	24	NEG	NEG	NEG	NEG	NEG	NEG
3	f	48	NEG	NEG	NEG	NEG	NEG	NEG
4	f	52	NEG	NEG	NEG	NEG	NEG	NEG
5	m	47	NEG	NEG	NEG	NEG	NEG	NEG
6	f	30	NEG	NEG	NEG	NEG	NEG	NEG
7	m	36	NEG	NEG	NEG	NEG	NEG	NEG
8	f	47	NEG	NEG	NEG	NEG	NEG	NEG
9	m	42	NEG	NEG	NEG	NEG	NEG	NEG
10	f	59	NEG	NEG	NEG	NEG	NEG	NEG
11	m	29	NEG	NEG	NEG	NEG	NEG	NEG
12	f	42	NEG	NEG	NEG	NEG	NEG	NEG
13	f	47	NEG	NEG	NEG	NEG	NEG	NEG
14	f	53	NEG	NEG	NEG	NEG	NEG	NEG
15	f	47	NEG	NEG	NEG	NEG	NEG	NEG
16	m	48	NEG	NEG	NEG	NEG	NEG	NEG
17	f	61	NEG	NEG	POS	POS	POS	POS
18	m	49	NEG	NEG	NEG	NEG	NEG	NEG
19	f	30	NEG	NEG	NEG	NEG	NEG	NEG
20	f	59	NEG	NEG	NEG	NEG	NEG	NEG
21	f	35	NEG	NEG	NEG	NEG	NEG	NEG
22	f	43	NEG	NEG	NEG	NEG	NEG	NEG
23	f	61	NEG	NEG	NEG	NEG	NEG	NEG
24	f	55	NEG	NEG	NEG	NEG	NEG	NEG
25	m	38	NEG	NEG	NEG	NEG	NEG	NEG
26	f	46	NEG	NEG	NEG	NEG	NEG	NEG
27	f	48	NEG	NEG	NEG	NEG	NEG	NEG
28	f	22	NEG	NEG	NEG	NEG	NEG	NEG
29	f	61	NEG	NEG	NEG	NEG	NEG	NEG
30	f	22	NEG	NEG	NEG	NEG	NEG	NEG
31	f	44	NEG	NEG	NEG	NEG	NEG	NEG
32	f	58	NEG	NEG	NEG	NEG	NEG	NEG
33	m	46	NEG	NEG	NEG	NEG	NEG	NEG
34	f	40	NEG	NEG	NEG	NEG	NEG	NEG
35	f	61	NEG	NEG	NEG	NEG	NEG	NEG
36	f	37	NEG	NEG	NEG	NEG	NEG	NEG

37	f	44	NEG	NEG	NEG	NEG	NEG	NEG
38	f	55	NEG	NEG	NEG	NEG	NEG	NEG
39	f	50	NEG	NEG	NEG	NEG	NEG	NEG
40	f	39	NEG	NEG	NEG	NEG	NEG	NEG
41	f	49	NEG	NEG	NEG	NEG	NEG	NEG
42	f	46	NEG	NEG	NEG	NEG	NEG	NEG
43	f	62	NEG	NEG	NEG	NEG	NEG	NEG
44	f	46	NEG	NEG	NEG	NEG	NEG	NEG
45	f	62	NEG	NEG	NEG	NEG	NEG	NEG
46	f	60	NEG	NEG	NEG	NEG	NEG	NEG
47	f	46	NEG	NEG	NEG	NEG	NEG	NEG
48	m	55	NEG	NEG	NEG	NEG	NEG	NEG
49	m	57	NEG	NEG	NEG	NEG	NEG	NEG
50	f	35	NEG	NEG	NEG	NEG	NEG	NEG
